# Accessory Proteins of the Nitrogenase Assembly, NifW, NifX/NafY, and NifZ, Are Essential for Diazotrophic Growth in the Nonheterocystous Cyanobacterium *Leptolyngbya boryana*

**DOI:** 10.3389/fmicb.2019.00495

**Published:** 2019-03-15

**Authors:** Aoi Nonaka, Haruki Yamamoto, Narumi Kamiya, Hiroya Kotani, Hisanori Yamakawa, Ryoma Tsujimoto, Yuichi Fujita

**Affiliations:** ^1^School of Agricultural Sciences, Nagoya University, Nagoya, Japan; ^2^Graduate School of Bioagricultural Sciences, Nagoya University, Nagoya, Japan

**Keywords:** cyanobacteria, nitrogen fixation, nitrogenase, MoFe protein, NifZ, NifW, NifX/NafY

## Abstract

Since nitrogenase is extremely vulnerable to oxygen, aerobic or micro-aerobic nitrogen-fixing organisms need to create anaerobic microenvironments in the cells for diazotrophic growth, which would be one of the major barriers to express active nitrogenase in plants in efforts to create nitrogen-fixing plants. Numerous cyanobacteria are able to fix nitrogen with nitrogenase by coping with the endogenous oxygen production by photosynthesis. Understanding of the molecular mechanisms enabling to the coexistence of nitrogen fixation and photosynthesis in nonheterocystous cyanobacteria could offer valuable insights for the transfer of nitrogen fixation capacity into plants. We previously identified the *cnfR* gene encoding the master regulator for the nitrogen fixation (*nif*) gene cluster in the genome of a nonheterocystous cyanobacterium *Leptolyngbya boryana*, in addition to initial characterization of the *nif* gene cluster. Here we isolated nine mutants, in which the *nif* and *nif*-related genes were individually knocked out in *L. boryana* to investigate the individual functions of (1) accessory proteins (NifW, NifX/NafY, and NifZ) in the biosynthesis of nitrogenase metallocenters, (2) serine acetyltransferase (NifP) in cysteine supply for iron-sulfur clusters, (3) pyruvate formate lyase in anaerobic metabolism, and (4) NifT and HesAB proteins. *ΔnifW*, *ΔnifXnafY*, and *ΔnifZ* exhibited the most severe phenotype characterized by low nitrogenase activity (<10%) and loss of diazotrophic growth ability. The phenotypes of *ΔnifX*, *ΔnafY*, and *ΔnifXnafY* suggested that the functions of the homologous proteins NifX and NafY partially overlap. *ΔnifP* exhibited significantly slower diazotrophic growth than the wild type, with lower nitrogenase activity (22%). The other four mutants (*ΔpflB*, *ΔnifT*, *ΔhesA*, and *ΔhesB*) grew diazotrophically similar to the wild type. Western blot analysis revealed a high correlation between nitrogenase activity and NifD contents, suggesting that NifD is more susceptible to proteolytic degradation than NifK in *L. boryana*. The phenotype of the mutants lacking the accessory proteins was more severe than that observed in heterotrophic bacteria such as *Azotobacter vinelandii*, which suggests that the functions of NifW, NifX/NafY, and NifZ are critical for diazotrophic growth of oxygenic photosynthetic cells. *L. boryana* provides a promising model for studying the molecular mechanisms that produce active nitrogenase, to facilitate the creation of nitrogen-fixing plants.

## Introduction

Nitrogen is an essential nutrient for all organisms, and its availability often limits plant productivity, for example, in cereals (Rosenblueth et al., 2018). Nitrogen fixation is a process by which atmospheric nitrogen (N_2_) is converted into ammonia (NH_3_), which is used by many organisms as a source of nitrogen.

The enzyme responsible for catalyzing the biological nitrogen fixation reaction is nitrogenase, which consists of two separable components: the Fe protein and the MoFe protein (Seefeldt et al., 2018). The Fe protein (a NifH dimer) catalyzes the ATP-dependent electron transfer reaction via a [4Fe-4S] cluster held in the interface between NifH protomers. The MoFe protein, serving as the catalytic component, has two metallocenters: the P-cluster (a [8Fe-7S] cluster) and the iron-molybdenum cofactor (FeMo-co; a [7Fe-9S-C-Mo-homocitrate] cluster). The electrons from the Fe protein are transferred to the P-cluster and, eventually, to FeMo-co, in which a nitrogen molecule is converted to two ammonia molecules. All three metallocenters are extremely vulnerable to oxygen. For example, upon exposure to air, the half-life of Fe protein holding the [4Fe-4S] cluster is only 30 s (Robson, 1979). In addition, FeMo-co is synthesized by a series of complex enzymatic reactions (Curatti and Rubio, 2014; Hu and Ribbe, 2016). In the first stage, a sulfur atom is released from Cys by Cys desulfurase (NifS), and a precursor cluster is assembled on NifU (Johnson et al., 2005). In the second stage, the NifB-cofactor (NifB-co) is formed by the action of NifB (Shah et al., 1994; Hernandez et al., 2007). In the third stage, the mature FeMo-co is assembled on the NifEN complex, and finally, the FeMo-co is transferred to the apo-form of the MoFe protein (Curatti et al., 2007; Kaiser et al., 2011; Fay et al., 2016). The intermediate clusters in the biosynthetic process of FeMo-co are also vulnerable to oxygen. Therefore, aerobic and micro-aerobic nitrogen-fixing organisms need to create strict anaerobic microenvironments in the cell to facilitate active nitrogenase functions.

Crop yields in current agriculture are heavily dependent on nitrogen fertilizer produced by industrial nitrogen fixation based on the Harbor-Bosch process. However, industrial nitrogen fixation consumes a lot of fossil fuel resulting in massive amounts of CO_2_ emissions, which contribute to global warming, and the application of nitrogen fertilizer in excess in crop fields causes serious environmental pollution. To alleviate the negative impacts of industrial nitrogen fixation without reducing the crop yield, novel technological innovations are awaited. One of the most promising innovations is the creation of nitrogen-fixing crops by transferring nitrogen fixation genes into plants (Curatti and Rubio, 2014; Burén and Rubio, 2018; Good, 2018). However, it is a challenging undertaking. A key obstacle is that nitrogenase should be protected not only from environmental oxygen but also from the endogenous oxygen produced by photosynthesis in crops. In addition, a number of genes, including the genes for nitrogenase cofactor biosynthesis, should be transferred into the plant genome and their expression should be appropriately regulated, which is a major additional obstacle.

The [4Fe-4S] cluster of Fe protein can be produced by the iron-sulfur cluster biosynthesis systems (ISC and SUF) of non-diazotrophic cells (Lopez-Torrejon et al., 2016). In contrast, special enzymes/proteins are required for the biosynthesis of P-cluster and FeMo-co in the MoFe protein. The special enzymes/proteins have been identified through molecular genetics and biochemical analyses in a limited number of heterotrophic bacteria such as *Azotobacter vinelandii* and *Klebsiella pneumoniae*. According to the current model of nitrogenase biosynthesis in the diazotrophs, other than the essential six proteins (NifHDKBEN), some accessory proteins are involved in the efficient biosynthesis of the metallocenters and their introduction to apo-forms of the MoFe protein (Curatti and Rubio, 2014; Burén and Rubio, 2018). NifZ is involved in the maturation of the P-cluster, which is formed by the reductive coupling of a pair of precursor [4Fe-4S] clusters. NifW was found to bind to an apo-form of the MoFe protein without FeMo-co, while its biochemical function is still unknown. The NifX and NafY proteins are involved in efficient transfer processes of NifB-co and FeMo-co to the NifEN and NifDK proteins, respectively. These are homologous proteins since the amino acid sequence of the C-terminal half of NafY exhibits high similarity to that of the entire NifX protein. While the *nifT*, *hesA*, and *hesB* genes are largely conserved in diazotrophic organisms, the functions of the proteins remain unknown.

Cyanobacteria are prokaryotes that perform oxygenic photosynthesis similar to plants. About half of cyanobacterial species can fix nitrogen (Stal and Zehr, 2008). Therefore, nitrogen-fixing cyanobacteria are a unique group of organisms in which oxygen-sensitive nitrogen fixation coexists with oxygen-producing photosynthesis. Some filamentous cyanobacteria such as *Anabaena* sp. PCC 7120 develop heterocysts, which are special nitrogen fixation cells, to spatially separate nitrogenase from photosynthesis (Herrero et al., 2016). However, some nonheterocystous nitrogen-fixing cyanobacteria exhibit nitrogenase activity in light conditions (Evans et al., 2000; Rabouille et al., 2006). Such cyanobacteria potentially have some unique systems for the biosynthesis and use of nitrogenase to cope with the endogenously produced oxygen. Elucidation of the molecular mechanisms could provide clues crucial to the mechanisms of functional expression of nitrogenase in plants.

The nonheterocystous cyanobacterium *Leptolyngbya boryana* offers a promising system for the investigation of the molecular mechanisms of functional expression of nitrogenase since a gene targeting technique has been established (Fujita et al., 1992; Tsujimoto et al., 2015) and the genome sequence is available (Hiraide et al., 2015). We have previously identified the nitrogen fixation (*nif*) gene cluster in *L. boryana*, wherein 50 *nif* and *nif*-related genes are clustered at the 50-kb chromosomal region. In the region, in addition to the structural genes of nitrogenase (*nifHDK*), there are numerous genes encoding proteins essential for the biosynthesis of FeMo-co (*nifBEN*) and iron-sulfur clusters (*nifSU*), accessory proteins (*nifWXZ*), ferredoxins (*fdx*), cytochrome *c* oxidase (*coxB2A2C2*), molybdenum transporter (*modABC*), transcriptional regulators (*cnfR* and *chlR*), and proteins with unknown functions (such as *nifT*, *hesAB*, and other open reading frames). Based on the phenotype of a mutant NK4 (*ΔcnfR*), in which *cnfR* was knocked out, we discovered that *cnfR* encodes the master transcriptional activator for the expression of *nif* genes in the *nif* gene cluster (Tsujimoto et al., 2014). In addition, we observed that four mutants, NK8, NK2, NK7, and NK9, in which chromosomal fragments carrying *nifX*, *nifZ-nifT*, *nifP-orf84-dpsA-orf99*, and *hesA-hesB-fdxH-feoA-fedB-mop*, respectively, were deleted, exhibited low nitrogenase activity and considerable growth defects under nitrogen fixation conditions. The phenotype of NK8 (*ΔnifX*) indicated that NifX is critical for nitrogenase activity and nitrogen-fixing growth. However, it remains unknown which gene is responsible for the phenotype in the three mutants.

*L. boryana* cells exhibit nitrogenase activity only under microoxic conditions. There are at least four genes (*pflA*, *pflB*, *adhE*, and *acs*) for anaerobic metabolism in the leftmost region of the *nif* gene cluster. *pflB* and *pflA* encode pyruvate formate lyase (PFL) and PFL activating enzyme (PFL-AE), respectively. PFL activated by PFL-AE catalyzes the conversion of pyruvate and CoA to acetyl-CoA and formate, playing a key role in anaerobic metabolism in *Escherichia coli*. However, it remains unknown whether PFL is critical in facilitating the nitrogenase activity and nitrogen fixation in cyanobacteria.

Here, we isolated eight *L. boryana* mutants, in which a single gene was deleted (*nifZ*, *nifT*, *nifP*, *nifW*, *hesA*, *hesB*, *pflB*, and *nafY*), and one *L. boryana* mutant, in which two homologous genes *nifX* and *nafY* were deleted. We evaluated them based on diazotrophic growth and nitrogenase activity, and classified them into four groups (Group 1 to 4). Particularly, the mutants of Group 1 (*ΔnifW*, *ΔnifX/nafY*, and *ΔnifZ*) exhibited much severer phenotype (no diazotrophic growth and low nitrogenase activity at less than 10%) than those of the relevant mutants in *A. vinelandii* and *K. pneumoniae*. The results suggest that the functions of NifW, NifX/NafY, and NifZ are critical for diazotrophic growth in oxygenic photosynthetic cells. The cyanobacterium *L. boryana* is a promising model photosynthetic organism for studying the molecular mechanisms that produce the active nitrogenase that facilitates diazotrophic growth and could facilitate efforts to create nitrogen-fixing plants.

## Materials and Methods

### Strains and Culture Conditions

The cyanobacterium *L. boryana* strain *dg5* (Fujita et al., 1996; Hiraide et al., 2015) was used as the wild type. NK1 (*ΔnifDK*), NK2 (*ΔnifZT*), and NK8 (*ΔnifX*) isolated previously (Tsujimoto et al., 2014) were used as the control strains. For growth or induction of the *nif* genes under microoxic conditions, agar plates were incubated in an anaerobic jar (BBL GasPak anaerobic systems; BD Biosciences) with a sachet to create anaerobic conditions (Gas Generating Kit Anaerobic System, Oxoid, Basingstoke, Hants, United Kingdom or AnaeroPack-Anaero; Mitsubishi Gas Chemical; Tokyo, Japan). As described previously (Tsujimoto et al., 2018), dry anaerobic indicator strips (Dry Anaerobic Indicator Strips, BD Biosciences) were used to confirm anaerobic conditions in the jar.

### Plasmid Construction

DNA fragments from *L. boryana* genomic DNA were amplified by PCR, using KOD FX Neo polymerase (Toyobo, Osaka, Japan), and separated by agarose electrophoresis to purify them from the excised agarose gel slice (Wizard SV Gel and PCR Clean-Up System, Sigma). After digestion with the appropriate restriction enzymes, the DNA fragments were ligated with an appropriate vector to construct a recombinant plasmid (DNA Ligation Kit, Mighty Mix, Takara, Kusatsu, Japan). To construct pNK75, we used the In-Fusion HD Cloning Kit (Takara). Detailed information on plasmid construction is provided in Supplementary Table 1.

### Transformation of *L. boryana*

To prevent single recombination between the plasmid and the chromosome in *L. boryana* cells, the plasmid was linearized by digestion with the appropriate restriction enzyme(s). The digested plasmid was introduced into *L. boryana* cells by electroporation, and the transformants were selected by kanamycin resistance (or chloramphenicol resistance for *ΔnafY*) on BG-11 agar plates containing kanamycin or chloramphenicol (Tsujimoto et al., 2015). Gene disruption in the isolated transformants was confirmed using colony PCR (Supplementary Figure 7 and Supplementary Table 2).

### Growth Comparison

Cells were grown under nitrate-replete (BG-11) and aerobic conditions for 2 days (50 μmol m^-2^ s^-1^) as pre-culture. The cells were subsequently suspended in sterile water to adjust the OD value to 1.0. An aliquot (6.0 μl) was spotted on new agar plates, BG-11 or BG-11_0_ (BG-11 without combined nitrogen), and incubated under microoxic conditions in the light (40 μmol m^-2^ s^-1^) for 5 days (Tsujimoto et al., 2014).

### Acetylene Reduction Assay

Nitrogenase activity was assayed as described previously (Tsujimoto et al., 2014). The cells grown on BG-11 under aerobic conditions for 2 days were suspended in water. Aliquots (300 μl with OD_730_ of 7.7) of the cell suspensions were spread uniformly to form a 4-cm diameter circle on a BG-11_0_ agar plate, which was then incubated in an anaerobic jar under continuous light (50 μmol m^-2^ s^-1^) conditions at 30°C for 16 h to induce the *nif* genes. After induction, the cells were harvested in liquid BG-11_0_ medium (1.5 ml with OD_730_ of 3). An aliquot (1.0 ml) of the suspension was transferred into a 5-ml glass vial (V-5A, Nichiden-rika glass, Kobe, Japan) and sealed tightly using a butyl rubber septum, and covered with an aluminum seal in the anaerobic chamber. The glass vials were purged with a gas mixture of 10% (vol/vol) acetylene in argon as the standard gas (Japan Fine Products, Kawasaki, Japan) for 45 s. The glass vials were incubated for 10 min under illumination (50 μmol m^-2^ s^-1^) at 30°C with stirring. The upper gas phase (500 μl) was analyzed using a gas chromatograph (GC-2014AF, Shimadzu, Kyoto, Japan) equipped with a Porapak N column (0.3 m × 3 mm, Shinwa Chemical Industries, Kyoto, Japan) using N_2_ as the carrier gas under isothermal conditions at 40°C. Ethylene was detected using a flame ionization detector. After the ethylene formation assay, the cells were collected to estimate optical density at 730 nm (OD_730_) using a V-550 spectrophotometer (JASCO, Hachioji, Japan).

### Preparation of RNA, RT-PCR, and Real-Time PCR

Total RNA samples were prepared as described (Tsujimoto et al., 2014). To synthesize cDNA, extracted RNA was converted to cDNA using ReverTra Ace (Toyobo) and oligo-dT primer. The synthesized cDNAs were amplified using SYBR Premix Ex Taq II (Takara) with primer sets for each target gene (Supplementary Table 2). qPCR reaction was performed using the StepOne^TM^ Plus Real-Time PCR System (Life technologies). As an internal control, the housekeeping gene, *rnpB*, which encodes the RNA subunit of RNase P, was used. Based on the comparative CT values, relative expression levels were calculated.

### Western Blot Analysis

The induced cells were harvested in 1.5 ml protein extraction buffer (50 mM HEPES-KOH; pH 7.5, 10 mM MgCl_2_) and the suspension’s cell density was adjusted to OD_730_ of 20. An aliquot (500 μl) of the suspension was subjected to cell disruption in a beads-beater-type homogenizer (BugCrusher GM-01, Taitec, Koshigaya, Japan) with glass beads (100 mg glass beads, 150–212 microns, Sigma) at 4°C. The resultant homogenates were centrifuged at 1,360 ×*g* for 3 min at 4°C to obtain the supernatant fraction. Protein concentration was determined using the Bradford assay (Protein Assay, Bio-Rad) with bovine serum albumin as the standard. Western blot analysis was carried out as described previously (Aoki et al., 2014). The NifH, NifD, and NifK proteins were detected using three antisera against NifH, NifD and NifK from *L. boryana*, respectively (Tsujimoto et al., 2018).

## Results

In the present study, we investigate the functional significance of the following proteins; (1) accessory proteins (NifW, NifX/NafY, and NifZ) for the biosynthesis of nitrogenase metallocenters, (2) Ser acetyltransferase (NifP) for Cys supply to the biosynthesis of iron-sulfur clusters of nitrogenase, (3) PFL (PflB) for anaerobic metabolism, and (4) proteins with unknown functions (NifT, HesA, and HesB). First, we confirmed the assignment of the eight genes by multiple alignments with amino acid sequences of *A. vinelandii* and other diazotrophic cyanobacteria (Supplementary Figures 1–5). In addition, we found another *nif*-related gene, LBDG_23680, outside the *nif* gene cluster that exhibited significant similarity to *nifY* and *nafY* (Supplementary Figure 2). LBDG_23680’s amino acid sequence is more similar to that of NafY (48.9%) than that of NifY (41.2%) in *A. vinelandii*. In addition, His134 and Cys138 are conserved in LBDG_23680. The conserved His (His121 of NafY in *A. vinelandii*) is critical for FeMo-co binding in NafY of *A. vinelandii* (Rubio et al., 2004). Therefore, we tentatively identified the gene as *nafY*. Semi-quantification of mRNA using RT-PCR revealed that constitutive expression of *nafY* was largely different from the *nifHDK* genes whose expression was observed only under nitrogen-fixing conditions (Supplementary Figure 6).

**FIGURE 1:**
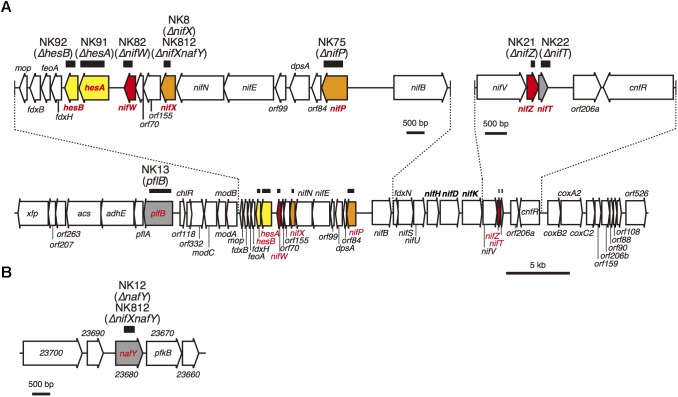
**(A)** Gene organization of the 50 kb nitrogen fixation gene cluster. **(B)** A chromosomal 6 kb region containing the *nafY* gene. The thick horizontal bars above the genes indicate the region that was removed and replaced with a kanamycin cartridge in the NK mutants. To illustrate the details, two chromosomal parts, from *nifB* to *mop* and from *nifV* to *cnfR* in the *nif* gene cluster, are enlarged. Based on the phenotype, the nine genes are color-coded as follows: red, genes essential for nitrogen fixing growth (Nif^-^ phenotype; Group 1); orange, genes critical for nitrogen fixing growth (Nif^S^ phenotype; Group 2); yellow, genes not essential for nitrogen fixing growth (Nif^+^) but lower nitrogenase activity (Group 3); and gray, genes not critical for nitrogen fixing growth (Nif ^+^) and normal nitrogenase activity (Group 4).

We isolated eight mutants in which each gene was individually knocked out (Figure 1). Since NifX exhibits considerably similarity with that of the C-terminal half of NafY (Supplementary Figure 2). We also isolated another mutant, in which both *nifX* and *nafY* genes were disrupted, to examine the functional redundancy of the homologous genes. Complete segregation of the knock-out copies from the wild type copies in the mutants was confirmed using colony PCR (Supplementary Figure 7).

**FIGURE 2:**
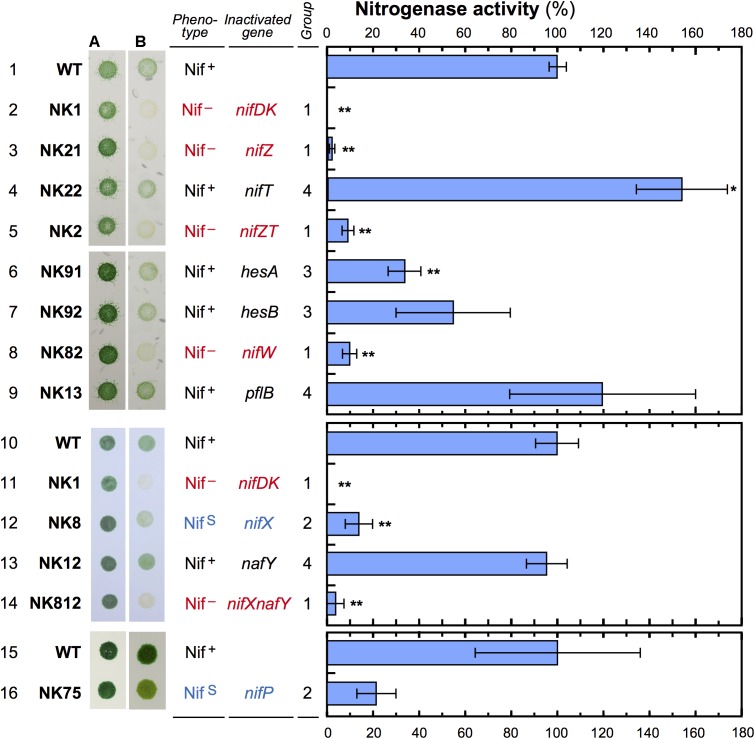
Comparison of growth and nitrogenase activity among the mutants. *L. boryana* cells were grown on BG-11 agar plates containing nitrate **(A)** or BG-11_0_ without combined nitrogen **(B)** under microoxic conditions for 5 days (light intensity, 40 μmol m^-2^ s^-1^). To determine nitrogenase activity, cells grown in nitrate-replete conditions were incubated under nitrogen fixation conditions for 16 h to induce the *nif* genes, and used for an acetylene reduction assay. Bars indicate standard deviation (*n* = 3). Nitrogenase activity was measured in three independent experimental sets. Nitrogenase activity in wild type was 80.5 (first set, lanes 1–9; *ΔnifZ*, *ΔnifT*, *ΔhesA*, *ΔhesB*, *ΔnifW*, and *ΔpflB*), 80.8 (second set, lanes 10–14; *ΔnifX*, *ΔnafY*, and *ΔnifXnafY*), and 28.6 (third set, lanes 15 and 16; *ΔnifP*) nmol ml^-1^h^-1^ OD_730_^-1^. Asterisks indicate statistically significant differences (^∗^*p* < 0.05; ^∗∗^*p* < 0.01), compared with values of the wild type in the respective experiments. In *ΔhesB* (lane 7) and *ΔnifP* (lane 16), *p*-values were 0.086 and 0.07681 compared with the wild type, respectively. *p*-value of *ΔnifP* is less than 0.05 when compared with the two other wild-type (lanes 1 and 10).

The isolated nine mutants were cultured on BG-11 and BG-11_0_ agar plates under microoxic conditions with the other three mutants, *ΔnifDK*, *ΔnifZT*, and *ΔnifX*, as controls (Figure 2A,B). Acetylene reduction activity of the cells was also assayed in three independent experimental sets. We classified the mutants into four groups (Group 1 to 4) based on diazotrophic growth and nitrogenase activity.

Three Group 1 mutants, *ΔnifZ*, *ΔnifW*, and *ΔnifXnafY*, did not exhibit substantially growth under nitrogen-fixing conditions (Nif^-^ phenotype). *ΔnifDK* and *ΔnifZT*, which were previously isolated, were contained in Group 1 (Tsujimoto et al., 2014). The Nif^-^ phenotype appears to be correlated with very low nitrogenase activity (less than 10% of the wild type level). Group 2 contained *ΔnifP* and *ΔnifX* (Tsujimoto et al., 2014), which exhibited a phenotype with slow growth (Nif^S^). Growth in *ΔnifP* was slightly better than that in *ΔnifX*, which is consistent with the higher nitrogenase activity in *ΔnifP* (22%) than that in *ΔnifX* (14%). Two Group 3 mutants, *ΔhesA* and *ΔhesB*, exhibited significantly lower nitrogenase activity (34 and 55 %, respectively) in the acetylene reduction assay, but they grew as well as the wild type. Three Group 4 mutants, *ΔnifT*, *ΔpflB*, and *ΔnafY*, did not exhibit diazotrophic growth defects under the experimental conditions, and they exhibited acetylene reduction activity comparable to that of the wild type or even higher than that of wild type.

The double mutant of *nifX* and *nafY* (*ΔnifXnafY*, Group 1) exhibited a Nif ^-^ phenotype with low activity (4%). Considering that the relevant single mutants, *ΔnifX* and *ΔnafY*, exhibited a Nif^S^ (Group 2) and a wild-type (Group 4) phenotype, respectively, the slow diazotrophic growth with low nitrogenase activity (14%) in *ΔnifX* could be facilitated by NafY action while the chromosomal location is outside of *nif* gene cluster. The *nafY* transcript levels were almost constant in the four examined conditions, and the transcript level was slightly higher in nitrogen-deficient conditions than in nitrate-replete conditions irrespective of microoxic and aerobic conditions (Supplementary Figure 6).

Notably, *ΔnifT*’s acetylene reduction activity was significantly higher than that of the wild type (Figure 2). The stimulatory effect was also observed in the double mutant *ΔnifZT* compared to the single mutant *ΔnifZ*.

PFL converts pyruvate to acetyl-CoA and formate, which may support nitrogen fixation under anaerobic conditions. However, the *ΔpflB* mutant exhibited normal diazotrophic growth with similar nitrogenase activity levels (Group 4), suggesting that the *pflB* gene is not essential for nitrogen fixation under the present conditions. In addition, *ΔpflB* and the wild type grew heterotrophically with nitrate as the N source under aerobic and dark conditions, while *ΔpflB*’s anaerobic heterotrophic growth in the dark was slightly lower than that of the wild type (Supplementary Figure 8).

**FIGURE 3:**
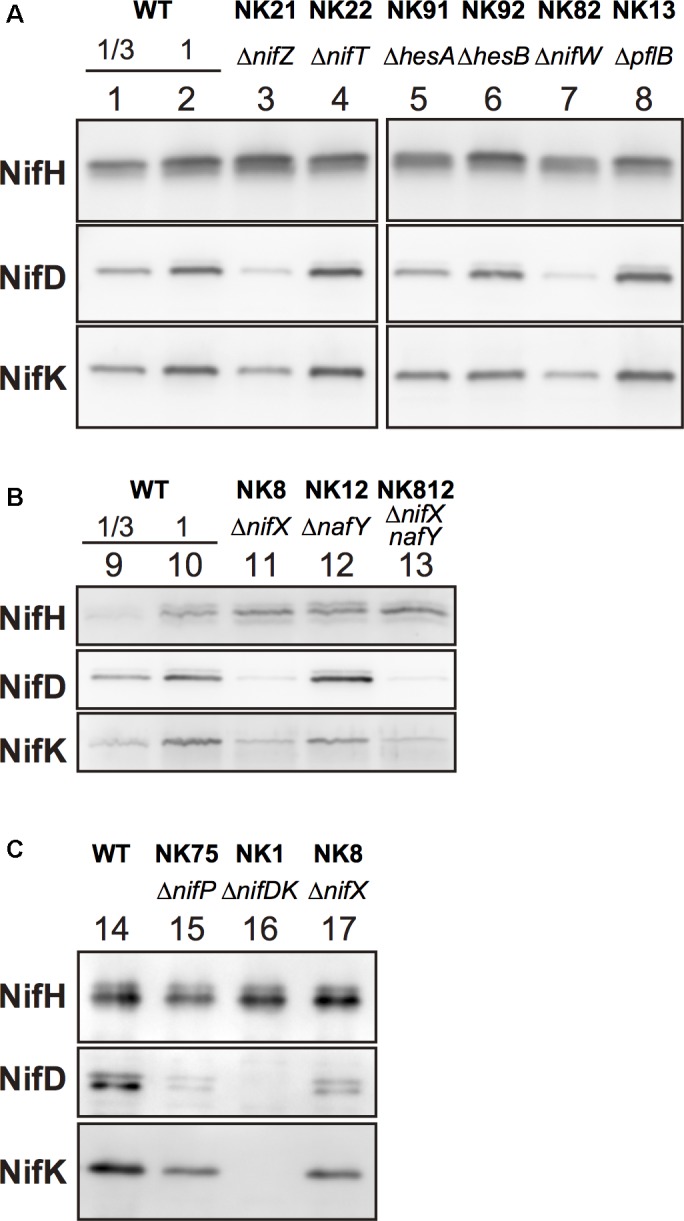
Western blot analysis of extracts of the mutants. Aliquots [**(A,C)**, 0.5 μg; and **(B)**, 1.5 μg] of total extracts from cells that induced the expression of the *nif* genes by incubation on BG-11_0_ under microoxic conditions for 16 h (24 h in **C**). For rough quantification, one-third of the protein amounts from the total extracts from the wild type cells were analyzed (lanes 1 and 9). The blotting membranes were the same in **(A)** (lanes 1–8). NifH, NifD, and NifK were detected by specific antisera (Tsujimoto et al., 2018).

To assess the effect of gene disruption on the amounts of nitrogenase subunits in the mutants, Western blot analysis was performed using specific antisera against individual nitrogenase subunits including NifH, NifD, and NifK (Figure 3). The NifH protein did not exhibit considerable change except in *ΔnifW*, in which it decreased marginally. NifD contents were the most drastically affected by mutations among the three subunits. They were markedly reduced in *ΔnifZ*, *ΔnifW*, *ΔnifX*, *ΔnifXnafY*, and *ΔnifP*, which showed good correlation with the nitrogenase activity levels (Figure 2). The NifK contents were also affected similar to NifD contents. However, the degree of decrease of NifK contents was less apparent compared to those of NifD. Although NifD and NifK’s signal intensities were almost similar to those in the wild type, NifD signals were much lower than those of NifK in *ΔnifZ*, *ΔnifW*, *ΔnifX*, *ΔnifXnafY*, and *ΔnifP*. The result suggests that NifD is more susceptible to proteolytic degradation than NifK in cyanobacterial cells when the MoFe protein is immature due to the absence of key accessory proteins.

## Discussion

In the present study, we isolated nine mutants in the nonheterocystous cyanobacterium *L. boryana* to investigate the targeted proteins′ physiological functions, particularly the role of nitrogenase assembly accessory proteins in cyanobacterial nitrogen fixation.

The kanamycin resistance (Km^R^) cartridge was used for target mutagenesis in *L. boryana* (Fujita et al., 1992, 1996, 1998; Okuhara et al., 1999; Kimata-Ariga et al., 2000; Tsujimoto et al., 2014; Hiraide et al., 2015). We observed that inserting the Km^R^ cartridge into a target gene forming an operon in the same direction as the transcription of the operon does not have a significant polar effect on the downstream genes. For example, the mutant YFD1, in which the Km^R^ cartridge was inserted in the intergenic region between the *chlL* and *chlN* genes forming an operon, there was no apparent phenotype (Kada et al., 2003), and we have been used YFD1 as a wild type showing Km^R^ (Kada et al., 2003; Yamazaki et al., 2006).

Considering numerous genes are closely clustered in the *nif* gene cluster, we investigated how the Km^R^ cartridge insertion influences downstream gene expression. In the *ΔnifP* mutant, the transcript level of *nifE*, which was located downstream of *nifP*, was quantified using real-time PCR and compared with that in the control strain YFD1 (Supplementary Figure 9). As a control, *nifH* transcript level, which is not affected by the Km^R^ cartridge insertion, was also quantified. The *nifE* transcript level in *ΔnifP* was approximately 75% of that in YFD1, and the transcript level of *nifH* in *ΔnifP* was also slightly lower than that in YFD1 (80%). The ratio of *nifE* to *nifH* was almost similar between *ΔnifP* (0.91) and YFD1 (1.0). The result suggests that the transcript level in *nifE* was marginally decreased by the insertion of the Km^R^ cartridge into the *nifP* coding region in *ΔnifP*, but the polar effect was not adequately strong to suppress the transcript level of a downstream gene, *nifE*, significantly. Therefore, we considered the polar effect of the Km^R^ cartridge insertion almost negligible in the *nif* gene cluster, although it was somewhat apparent.

The contents of MoFe protein, particularly NifD, decreased substantially, in Group 1 and 2 mutants, suggesting that the proteins, namely NifZ, NifW, NifX/NafY, NifP, are mainly involved in the maturation processes of the MoFe protein including the biosynthesis of FeMo-co and the formation of the P-clusters. Nitrogenase biosynthesis, including the MoFe protein, has been studied mostly in heterotrophic bacteria such as *A. vinelandii* and *K. pneumonia*. NifZ is involved in P-cluster maturation (Hu et al., 2007). While NifW’s biochemical function remains largely unknown, NifW is associated with an apo-form of the MoFe protein which carries immature P-clusters without FeMo-co (Jimenez-Vicente et al., 2018). Interactions between NifW and NifZ have previously been reported (Lee et al., 1998). NifX is involved in the transfer of the NifB-co, produced by NifB, to the scaffold protein NifEN (Hernandez et al., 2007). NafY is associated with another apo-form of the MoFe protein that has the P-cluster but not FeMo-co (Jimenez-Vicente et al., 2018), suggesting that NafY is involved in the efficient transfer and insertion of the FeMo-co in the MoFe protein.

Despite the potentially critical biochemical functions of accessory proteins during the maturation processes of MoFe protein, mutants that lacked the genes exhibited only weak or no phenotypes in *A. vinelandii* and *A. chroococcum* (Jacobson et al., 1989b; Paul and Merrick, 1989), which suggests that NifZ, NifW, NifX, and NafY are not essential for the production of active MoFe protein, and they may be required for efficient active MoFe protein production. The conditions under which the accessory proteins are required are unknown. However, NafY is thought to play important roles under molybdenum-deficient conditions (Rubio et al., 2002). In contrast, relevant cyanobacterial mutants showed much more severe phenotypes than heterotrophic bacteria, suggesting that NifZ, NifW, and NifX/NafY proteins play critical roles in production of active MoFe protein in cyanobacteria, which produce oxygen by photosynthesis. The accessory proteins may be critical in solving the oxygen paradox between nitrogen fixation and photosynthesis.

Cyanobacteria performing oxygenic photosynthesis may have some unique features during the nitrogenase biosynthesis. For example, *nifM* and *nifQ* are missing from diazotrophic cyanobacteria genomes, suggesting that the active Fe protein is assembled without NifM and that molybdenum is incorporated into the FeMo-co without NifQ in cyanobacteria (Tsujimoto et al., 2018). In addition, while the NafH protein is associated with the apo-form of MoFe protein in *A. vinelandii* (Jimenez-Vicente et al., 2018), the corresponding gene, *nafH*, has not been found in most diazotrophic cyanobacterial genomes including that of *L. boryana*.

We found the *nafY* gene outside the *nif* gene cluster in the *L. boryana* genome, and its transcription was largely constitutive (Supplementary Figure 6). Such features of the *nafY* gene are similar to those in *A. vinelandii*, in which the *nafY* mRNA was detected in cells grown with ammonia as well as in cells derepressed for nitrogen fixation (Rubio et al., 2002). While the single *nafY* mutant *ΔnafY* grew well, exhibiting normal nitrogenase activity, and Δ*nifX* retained diazotrophic growth capacity with very low nitrogenase activity (14%), the double mutant Δ*nifXnafY* lost all diazotrophic growth ability, exhibiting only 4% activity in *L. boryana*. Such phenotypic features suggest that the function(s) of the two homologous proteins, NifX and NafY, partially overlap and NafY slightly complements NifX’s function in cyanobacteria. The result seems to be consistent with the observation in *A. vinelandii*, in which *ΔnafYnifX* mutant showed poor diazotrophic growth and very low nitrogenase activity under stress conditions where molybdate was not supplemented (Rubio et al., 2002). It may imply that the standard cyanobacterial diazotrophic condition corresponds to the stress condition in *A. vinelandii*. A biochemical analysis is required to reveal further insights on the Nif-related accessory proteins in *L. boryana*.

The *nifP* gene [or *cysE* or *nafG* (Jimenez-Vicente et al., 2018)] encodes Ser acetyltransferase (SAT), which catalyzes the conversion of Ser to *O*-acetylserine, the direct precursor of Cys. CysM/CysK (*O*-acetylserine sulfhydrylase) converts *O*-acetylserine to Cys, and NifS liberates the sulfur atom of Cys for the iron-sulfur cluster assembly. The *nif*-specific SAT is dispensable during diazotrophic growth in *A. vinelandii* (Jacobson et al., 1989a). In contrast, the severe *ΔnifP* phenotype in *L. boryana* suggests that NifP contributes to Cys production under nitrogen fixation conditions, to produce nitrogenase carrying many iron-sulfur clusters. To satisfy sulfur atoms’ high demand for Fe protein and for MoFe protein biosynthesis, the additional Cys production by NifP could be important, in addition to the CysE (encoded by LBDG_53060), which is constitutively expressed as a housekeeping enzyme (Supplementary Figure 3). It is notable that 32 Cys molecules are required to produce one [4Fe-4S] cluster (the Fe protein) and pairs of P-clusters and FeMo-co (the MoFe protein), which are slightly more than what are required by Cys molecules (30 molecules) for the polypeptide parts of the Fe protein (NifH)_2_ and the MoFe protein (NifD-NifK)_2_ (5, 6, and 4 Cys residues in NifH, NifD, and NifK, respectively). Considering the rapid turnover (with a half-life of 5 min) of the [4Fe-4S] cluster of the Fe protein in *A. vinelandii* (Curatti et al., 2005), the Cys demand would be much greater than such a static estimation.

The *hesA* and *hesB* genes are conserved in diazotrophic cyanobacteria genomes, but their functions remain unknown. *E. coli* transformants exhibiting nitrogenase activity were isolated by the overexpression of the *nif* and *nif*-related genes from a gram-positive soil bacterium, *Paenibacillus* sp. WLY78 (Wang et al., 2013). The *hesA* gene is contained in the *nif* gene cluster of *Paenibacillus* sp. An *E. coli* transformant, harboring the *nif* gene cluster without *hesA*, showed approximately 60% of the nitrogenase activity of the transformant harboring the intact *nif* gene cluster. In *L. boryana*, nitrogenase activity was reduced to 34% in *ΔhesA*, and the decreased nitrogenase activity was well correlated with the decrease in NifDK proteins. The result suggests that HesA is involved in the efficient production of the MoFe protein but is not essential in cyanobacteria.

Deletion of *nifT* does not affect diazotrophic growth in *K. pneumonia* and *A. vinelandii* (Jacobson et al., 1989a; Simon et al., 1996). However, notably, the *L. boryana* *ΔnifT* mutant had nitrogenase activity that was significantly higher than that of the wild type. The trend was also observed in *ΔnifZT*. The NifT protein may have some suppressive effects on nitrogenase. More biochemical studies are required to determine the functions of NifT in cyanobacteria.

The *pflB* gene encodes PFL, which is involved in anaerobic carbon metabolism along with CoA-linked acetaldehyde dehydrogenase-alcohol dehydrogenase (encoded in *adhE*) and acetyl-CoA synthase (encoded by *acs*). In the unicellular cyanobacterium *Synechococcus* OS-B’ growing in the microbial mat of the Octopus Spring in Yellowstone National Park, the *pflB* gene was mainly expressed in the night time along with *nifHDK* genes, suggesting that the anaerobic metabolism supports energy production to facilitate nitrogenase activity in the dark (Steunou et al., 2006). However, the *ΔpflB* mutant did not show any phenotype under the nitrogen fixation conditions, although its heterotrophic growth under anaerobic conditions in the dark was slightly slower than that of the wild type (Supplementary Figure 8). To clarify the physiological functions of the enzyme during cyanobacterial nitrogen fixation, growth of *ΔpflB* under various diazotrophic conditions, such as low light, complete darkness, or the presence of glucose should be examined.

In addition to the target gene disruption technique, we recently developed an *in vivo* transposon tagging system to isolate random mutants in *L. boryana* (Tomatsu et al., 2018). The technique may facilitate the identification of novel genes that are not in the *nif* gene cluster of *L. boryana* or in the genomes of heterotrophic bacteria such as *A. vinelandii*. Further studies focusing on the molecular mechanisms underlying nitrogen fixation coexistence with photosynthesis are underway in our laboratory. Such studies using diazotrophic cyanobacteria may provide a molecular basis that could facilitate the creation of nitrogen-fixing plants in the future (Tsujimoto et al., 2018).

## Data Availability

All datasets generated for this study are included in the manuscript and/or the Supplementary Files.

## Author Contributions

YF and RT conceived the study and designed the experiments. NK performed RT-PCR of *nifX* and *nafY*, and isolated *ΔnifX* and *ΔnafY* to perform initial characterization. AN, HK, and RT isolated all other mutants and characterized them except for *ΔnifP*. HaY isolated *ΔnifP* and characterized it. YF, AN, RT, and HiY wrote the manuscript. All authors reviewed the manuscript.

## Conflict of Interest Statement

The authors declare that the research was conducted in the absence of any commercial or financial relationships that could be construed as a potential conflict of interest.
