# Supplementary material for: Accessory Proteins of the Nitrogenase Assembly, NifW, NifX/NafY, and NifZ, Are Essential for Diazotrophic Growth in the Nonheterocystous Cyanobacterium Leptolyngbya boryana
Source: Front Microbiol. 2019 Mar 15;10:495. doi: 10.3389/fmicb.2019.00495 (PMC6428710; doi:10.3389/fmicb.2019.00495)
Supplement: Supplementary file 1 [file Data_Sheet_1.docx]

Supplementary Material

Accessory proteins of the nitrogenase assembly, NifW, NifX/NafY, NifZ, are essential for diazotrophic growth in the nonheterocystous cyanobacterium *Leptolyngbya boryana*

**Aoi Nonaka^1^, Haruki Yamamoto^2^, Narumi Kamiya^1^, Hiroya Kotani^2^, Hisanori Yamakawa^2^, Ryoma Tsujimoto^2^, and Yuichi Fujita^1,2*^**

*** Correspondence:** Dr. Yuichi Fujita: fujita@agr.nagoya-u.ac.jp

# Supplementary Figures and Tables

## Supplementary Figures


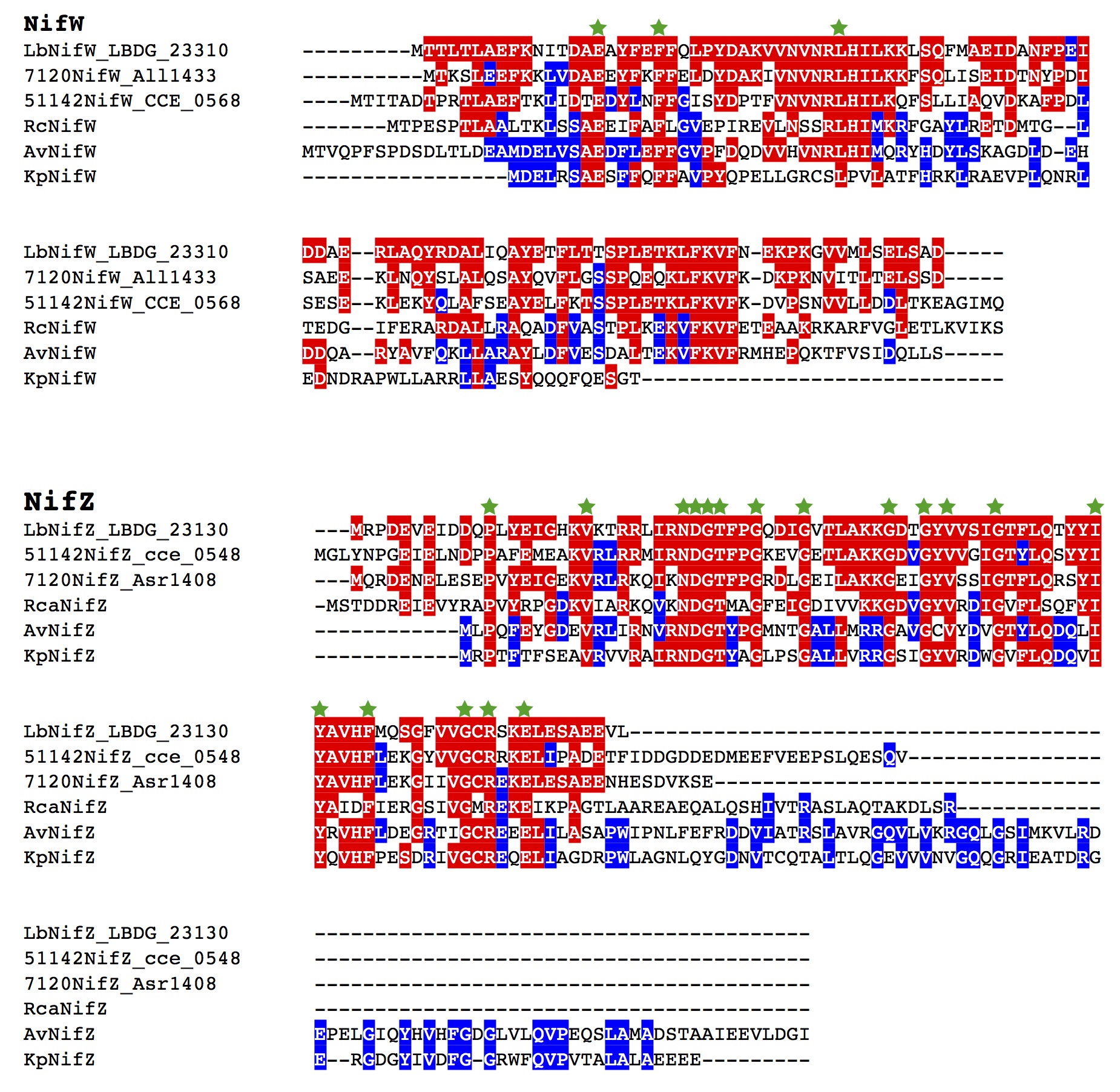


## Supplementary Figure 1. Multiple sequence alignments of NifW and NifZ

## LbNifW_LBDG_23310, NifW from *L. boryana*; 7120NifW_All1433, NifW from *Anabaena* sp. PCC 7120; 51142NifW_CCE_0568, NifW from *Cyanothece* sp. ATCC 51142; RcaNifW, NifW from *Rhodobacter capsulatus*; AzNifW, NifW from *A. vinelandii*; KpNifW, NifW from *K. pneumoniae*. LbNifZ_LBDG_23130, NifZ from *L. boryana*; 51142NifZ_cce_0548, NifZ from *Cyanothece* sp. ATCC 51142; 7120NifZ_Asr1408, NifZ from *Anabaena* sp. PCC 7120; RcaNifZ, NifZ from *R. capsulatus*; AvNifZ, NifZ from *A. vinelandii*; KpNifZ, NifZ from *K. pneumoniae*. Amino acid residues identical to NifW/NifZ from *L. boryana* are shown with a red background, and those identical to NifW/NifZ from *A. vinelandii* but not *L. boryana* are shown with a blue background. Amino acid residues conserved among all NifW and NifZ are indicated by green stars.


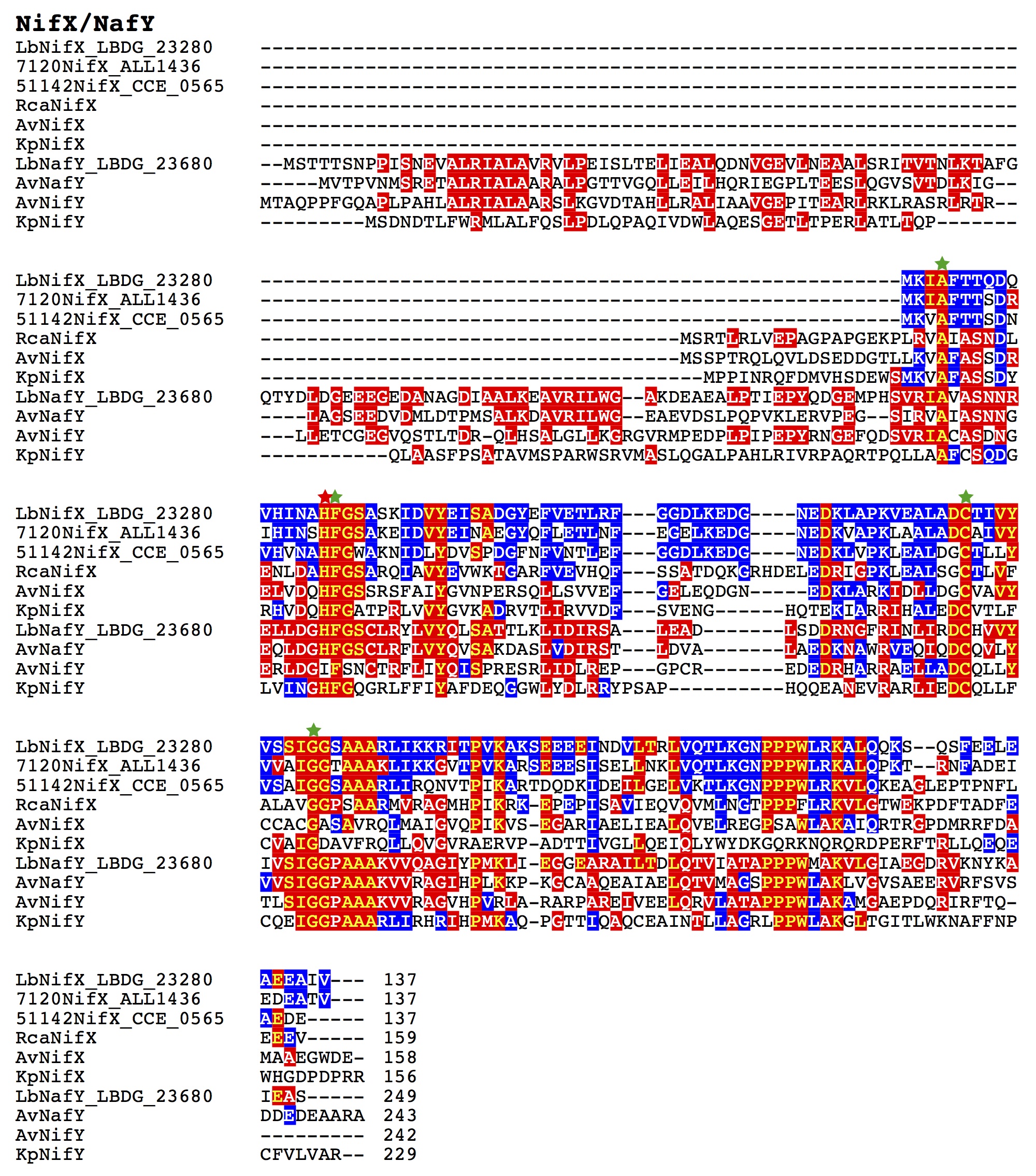


## Supplementary Figure 2. Multiple sequence alignments of NifX/NifY/NafY

## LbNifX_LBDG_23280, NifX from *L. boryana*; 7120NifX_ALL1436, NifX from *Anabaena* sp. PCC 7120; 51142NifX_CCE_0565, NifX from *Cyanothece* sp. ATCC 51142; RcaNifX, NifX from *R. capsulatus*; AvNifX, NifX from *A. vinelandii*; KpNifX, NifX from *K. pneumoniae*; LbNafY_LBDG_23680, NafY from *L. boryana*; AvNafY, NafY from *A. vinelandii*; AvNifY, NifY from *A. vinelandii*; and KpNifY, NifY from *K. pneumoniae*. Amino acid residues identical to NifX and NafY from *L. boryana* are shown in blue and red backgrounds, respectively. Those conserved between NifX and NafY are shown in yellow letters with a red background. Four amino acid residues, which are conserved among all proteins, are indicated by green stars, and the conserved His residue critical for FeMo-co binding (Rubio et al. 2004) is shown with a red star.


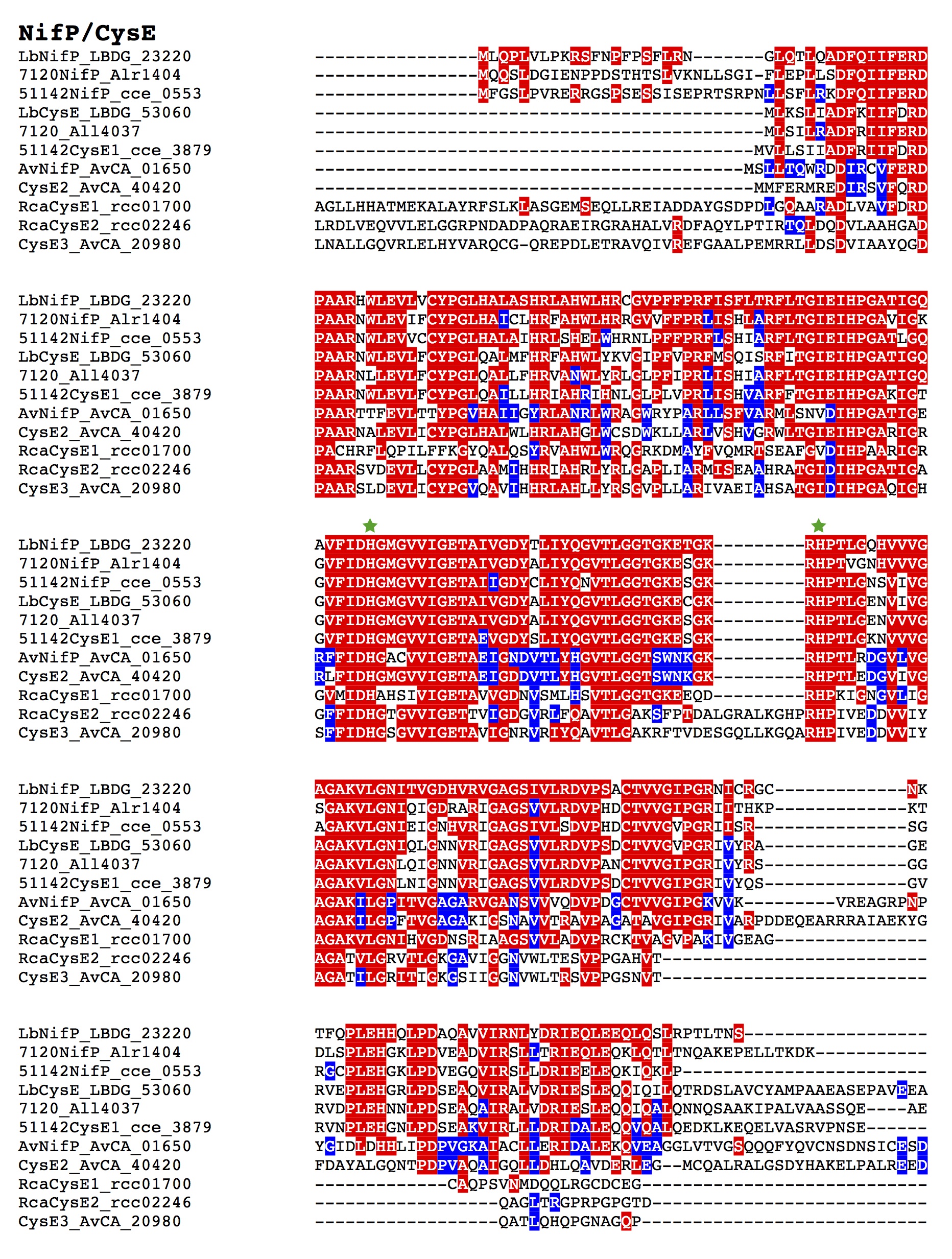


## Supplementary Figure 3. Multiple sequence alignments of NifP/CysE

LbNifP_LBDG_23220, NifP from *L. boryana*; 7120NifP_Alr1404, NifP from *Anabaena* sp. PCC 7120; 51142NifP_cce_0553, NifP from *Cyanothece* sp. ATCC 51142; LbCysE_LBDG_53060, CysE from *L. boryana*; 7120NifP_All4037, CysE from *Anabaena* sp. PCC 7120; 51142CysE1_cce_3879, CysE1 from *Cyanothece* sp. ATCC 51142; AvNifP_AvCA_01650, NifP from *A. vinelandii*; CysE2_AvCA_40420, CysE from *A. vinelandii*; RcaCysE1_rcc01700, CysE1 from *R. capsulatus*; RcaCysE2_rcc02246, CysE2 from *R. capsulatus*; CysE3_AvCA_20980, CysE3 from *A. vinelandii*. Amino acid residues identical to NifP from *L. boryana* are shown with a red background, and those identical to NifP from *A. vinelandii* but not to that from *L. boryana* are shown with a blue background. Green stars indicate two His residues that are important for the binding of Ser in *E. coli* (Pyre et al. 2004).


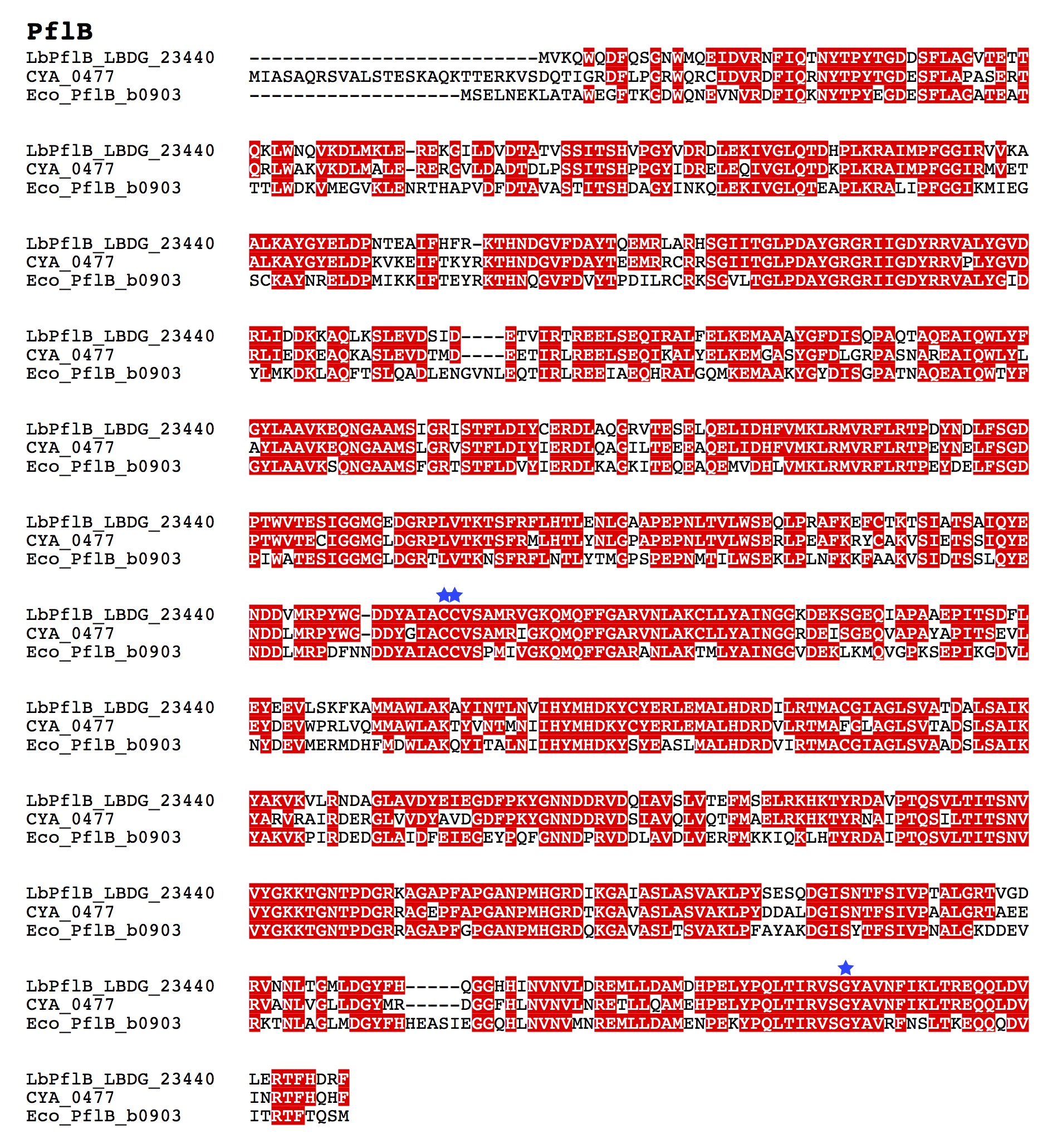


## Supplementary Figure 4. Multiple sequence alignments of PflB

## LbPflB_LBDG_23440, PflB from *L. boryana*; CYA_0477, PflB from Cyanobacteria Yellowstone A’; Eco_PflB_b0903, PflB from *E. coli*. Amino acid residues conserved among three PflBs are shown with a red background. Gly734, Cys418, and Cys419 indicated by blue stars constitute the active sites of PFL from *E. coli* (Becker et al. 1999). A glycyl radical is formed on Gly734 by the electron transfer from PFL-AE.


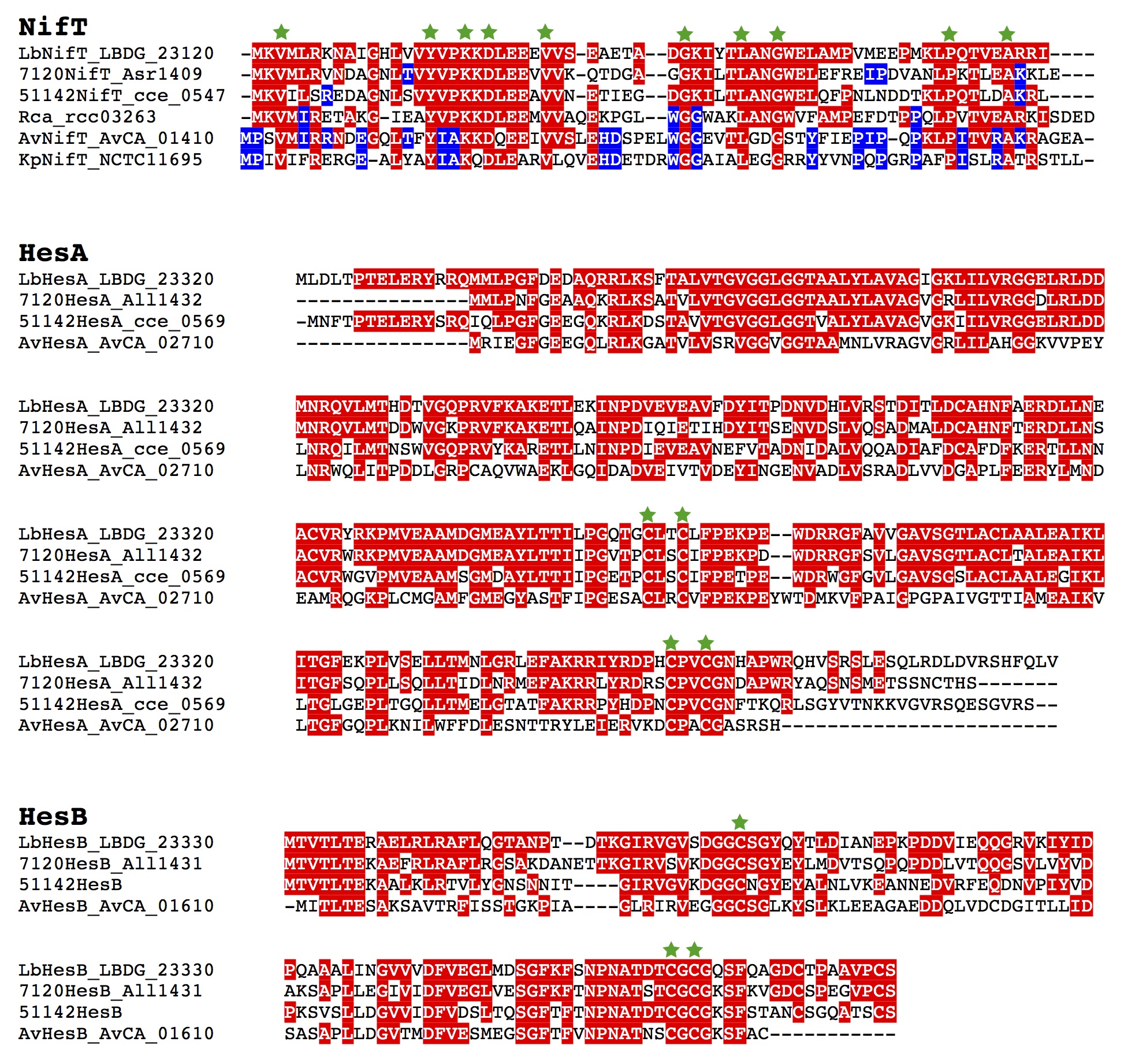


## Supplementary Figure 5. Multiple sequence alignments of NifT, HesA, and HesB

## LbNifT_LBDG_23120, NifT from *L. boryana*; 7120NifT_Asr1409, NifT from *Anabaena* sp. PCC 7120; 51142NifT_cce_0547, NifT from *Cyanothece* sp. ATCC 51142; Rca_rcc03263, NifT from *R. capsulatus*; AzNifT_AvCA_01410, NifT from *A. vinelandii*; KpNifT_NCTC11695, NifT from *K. pneumoniae*. LbHesA_LBDG_23320, HesA from *L. boryana*; 7120HeaA_All1432, HesA from *Anabaena* sp. PCC 7120; 51142HesA_cce_0569, HesA from *Cyanothece* sp. ATCC 51142; AvHesA_AvCA_02710, HesA from *A. vinelandii*. LbHesB_LBDG_23330, HesB from *L. boryana*; 7120HeaB_All1431, HesB from *Anabaena* sp. PCC 7120; 51142HesB, HesB from *Cyanothece* sp. ATCC 51142; AvHesB_AvCA_01610, HesB from *A. vinelandii*. In the NifT alignment, amino acid residues identical to NifT from *L. boryana* are shown with a red background, and those identical to NifT from *A. vinelandii* but not to that from *L. boryana* are shown with a blue background. Amino acid residues conserved among all NifT are shown by green stars. In the HesA and HesB alignments, amino acid residues identical to HesA and HesB from *L. boryana* are shown with a red background. Four and three Cys residues that are conserved among all HesA and HesB, respectively, are shown using green stars.


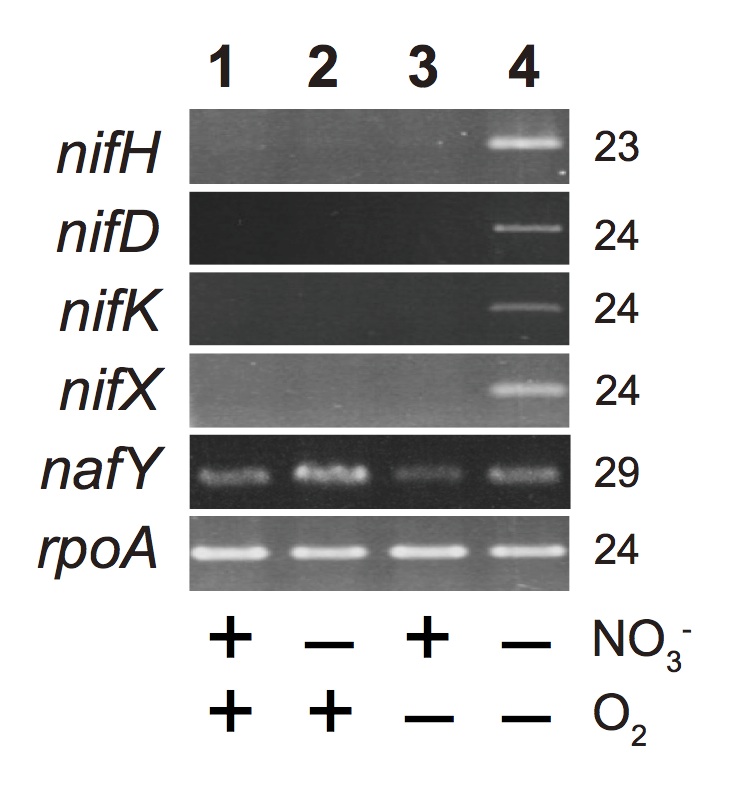


**Supplementary Figure 6.** Semi-quantification of transcripts of *nifX* and *nafY*. cDNA was amplified with primers shown in Supplementary Table 2. RT-PCR cycle numbers are shown on the right sides. The cells were incubated under aerobic and nitrate-replete (lane 1), aerobic and nitrate-depleted (lane 2), microoxic and nitrate-replete (lane 3), and microoxic and nitrate-depleted (lane 4) conditions.


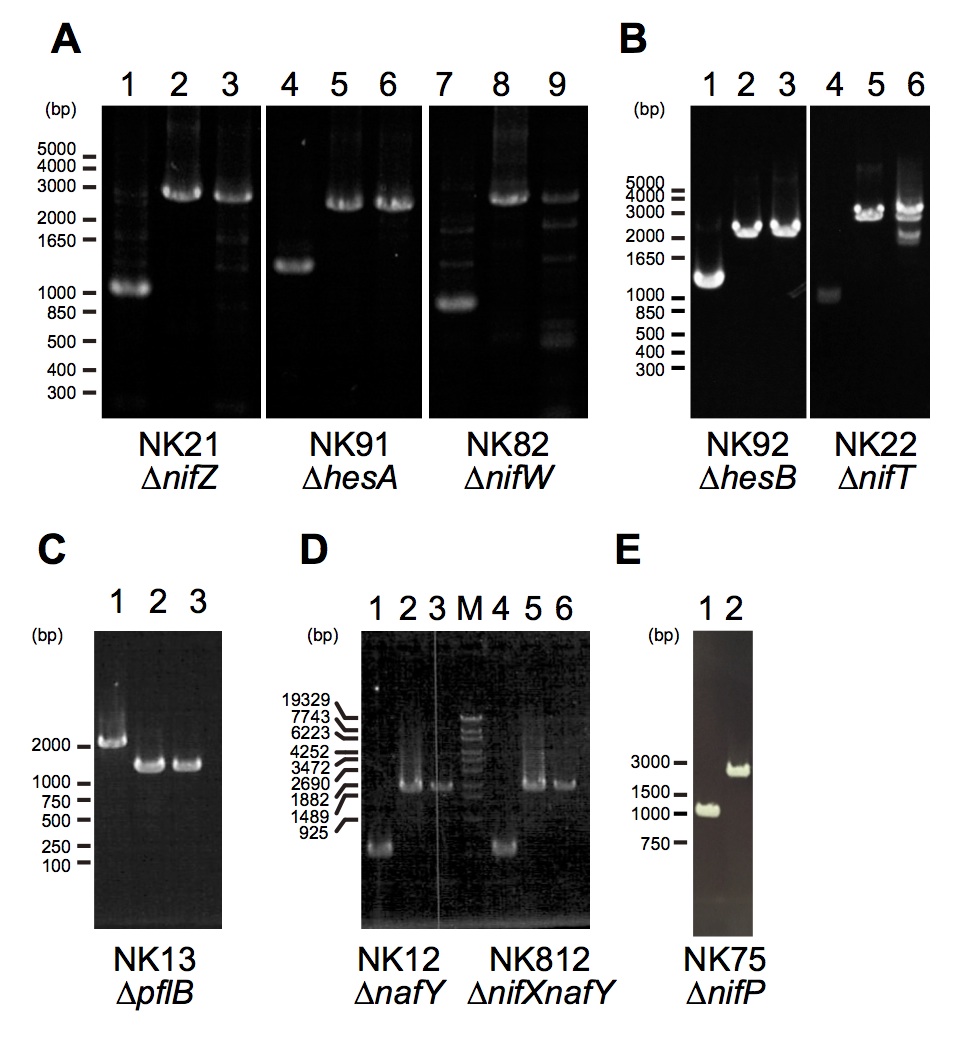


**Supplementary Figure 7.** Colony PCR to confirm complete segregation of mutant genomes. A. Lanes 1, 4, and 7: wild type; lanes 2, 5, and 8: plasmid used for transformation; and lanes 3, 6, and 9: *∆nifZ*, *∆hesA*, and *∆nifW*, respectively. B. Lanes 1 and 4: wild type; lanes 2 and 5: plasmid used for transformation; and lanes 3 and 6: *∆hesB* and *∆nifT*, respectively. C. Lane 1, wild type; lane 2, plasmid used for transformation; and lane 3, *∆pflB*. D. Lanes 1 and 4, wild type: lanes 2 and 5: plasmid used for transformation; and lanes 3 and 6: *∆nafY* and *∆nifXnafY*, respectively. E. Lane 1, wild type; lane 2, *∆nifP*. Primers are listed in Supplementary Table 1. In *∆pflB*, three primers were used. The size of the wild type and the mutant PCR products were almost similar when using two normal primers. Therefore, we used one common primer PbpflA-r1 and two primers, PbpflB-f5 and KmR-f1, which anneal to the *pflB* coding region that was removed in the mutant and the Km^R^ cartridge, respectively.


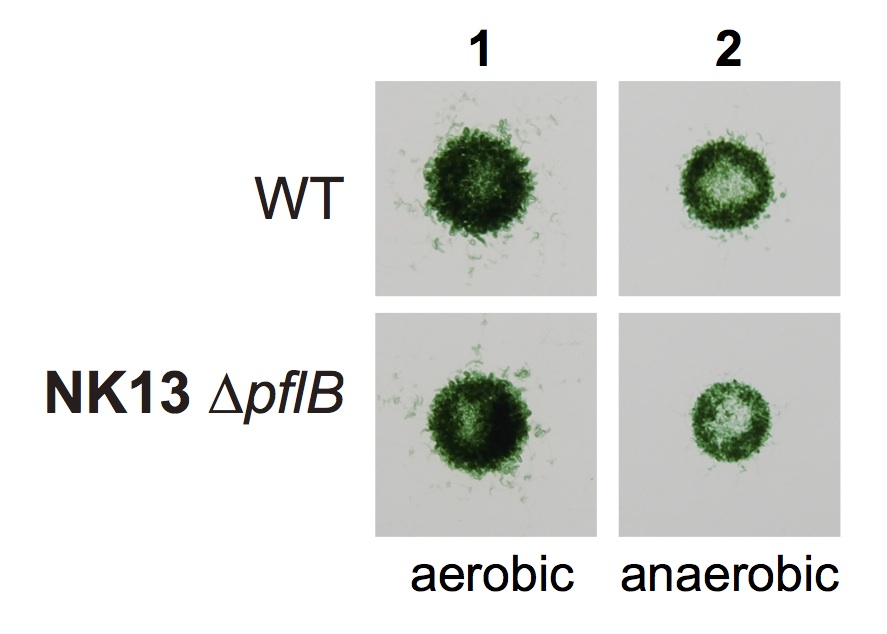


**Supplementary Figure 8.** Growth comparison of *∆pflB* in heterotrophic conditions (BG-11 containing 30 mM glucose). Wild type and *∆pflB* were grown under aerobic (lane 1) and anaerobic (lane 2) conditions in the dark for 14 days.


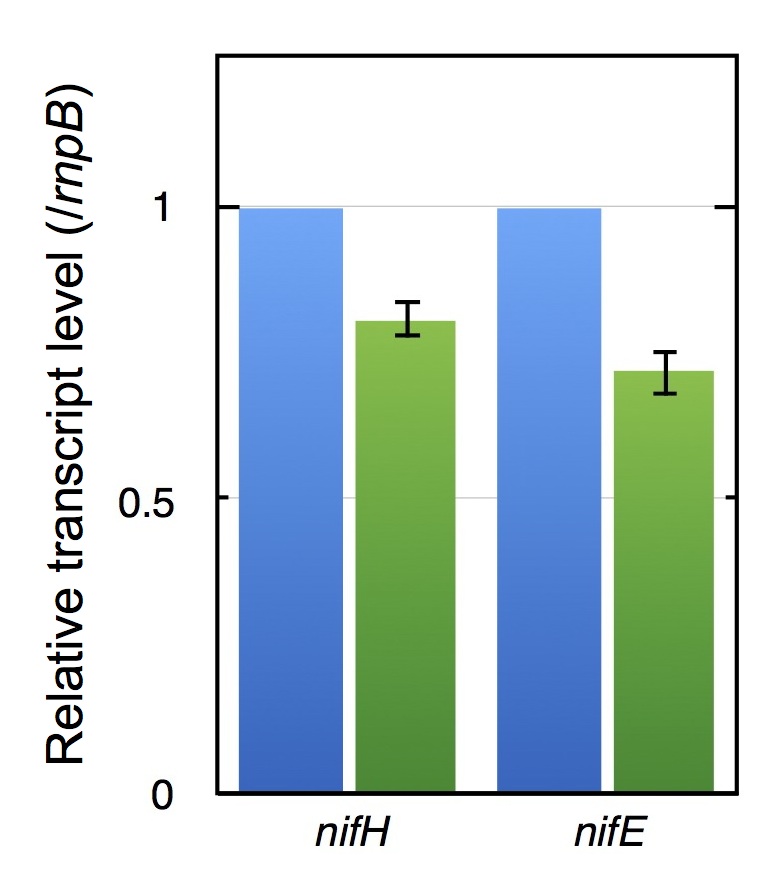


**Supplementary Figure 9.** Real-time PCR for quantification of transcripts of *nifE* in a control strain YFD1 (blue) and *∆nifP* (green). Total RNA was prepared from cells incubated under nitrogen fixation conditions as the method of measuring nitrogenase activity. The transcript levels of *nifH* and *nifE* relative to *rnpB* in YFD1 were normalized to 1.0.

## Supplementary Tables

**Supplementary Table 1** List of primers used for the construction of plasmids to isolate knock-out mutant

| Plasmid^a^ | Target gene | 5′/3′^b^ | D^c^ | Primer | Sequence^d^ | Length (bp)^e^ | Plasmid, Cloning site^f^ | Restriction Enzyme(s)^g^ |
| --- | --- | --- | --- | --- | --- | --- | --- | --- |
| pNK21 | *nifZ* | 5′ | F | PbnifK-f2SacXho | CGAAGGAGCTCGAGACGAACGGGGA | 1,843 | pUCK192^h^, SacI | SalI, XhoI |
|  |  |  | R | PbnifZ-r1Sacl | GAGTTACACCGAGCTCTTGTCCTGG |  |  |  |
|  |  | 3′ | F | PbnifZ-f2SalI | CATTTTATGCAGTCGACGGTTTTGTAGTC | 1,600 | pNK2^h^, SalI |  |
|  |  |  | R | PbpatB-f6XhoI | GCTTGCTCGAGCGGGCTGGAAAG |  |  |  |
| pNK22 | *nifT* | 5′ | F | PbnifK-f2SacXho | CGAAGGAGCTCGAGACGAACGGGGA | 2,046 | pNK2^h^, SacI | XhoI |
|  |  |  | R | PbnifT-r5SacI | CTTCTTCGAGCTCATAAACAACCAAATG |  |  |  |
| pNK71^†^ | *nifP* | 5′ | F | PbnifP-f2XhoI | CGAACAGCTCGAGGAGCAACTGC | 1,790 | pNK7^h^, SalI | na |
|  |  |  | R | PbnifE-r5SalI | GATCAGTGCAGTCGACAAGTCGAATAG |  |  |  |
| pNK75^§^ |  | 5′ | F | PbnifP-79-f | CCAGAGCGATCTCCGAATCAGCATTTTG | 197 | In-Fusion | SalI, XhoI |
|  |  |  | R | PbnifP+118-r | ACCGAGCTGCCGCAGGATCACGTTCAAAG |  |  |  |
|  |  | 3′/v | F | PbnifP+118-f | CCTGCGGCAGCTCGGTACCTGTTCACTG | – |  |  |
|  |  |  | R | PbnifP-79-r | TTCGGAGATCGCTCTGGATAGGATTTAT |  |  |  |
| pNK91 | *hesA* | 3′ | F | PbhesA-f3XhoI | GAGTCACAACTCGAGATTTAGATGTGC | 1,632 | pNK9^h^, SalI | SalI, XhoI |
|  |  |  | R | Pbmop-r5SalI | AGCTAAGCCGTCGACTGAGGATTTCG |  |  |  |
| pNK92 | *hesB* | 3′ | F | PbhesB-f4XhoI | CTTTCCAAGCTCGAGATTGCACTCC | 1,778 | pUCK192^h^, SalI | na |
|  |  |  | R | PbmodA-r2SalI | CAAGTTGCCCGGTCGACCGAAGTTC |  |  |  |
|  |  | 5′ | F | Pborf155-f2BamHI | CCTTGCGGGATCCTCAACGATTTGG | 2,010 | BamHI* | KpnI, SalI |
|  |  |  | R | PbhesB-r4BamHI | CGTATCTGTAGGATCCGCTGTGCCTTG |  |  |  |
| pNK80^†^ | *nifX-nifW* | 5′ | F | PbnifE-f3SacXho | GATGAGAGCTCGAGCACCTTATCGAGC | 1,939 | pUCK192^h^, SacI | na |
|  |  |  | R | PbnifX-r1Sacl | TCTTTGAGCTCACCCCCAAATCTCAAC |  |  |  |
|  |  | 3′ | F | PbnifW-f3Sall | TCGCTGAGTGTCGACTCTCTGTCTTGC | 1,788 | XhoI* | na |
|  |  |  | R | PbfdxH-r3Xhol | CAGGAAGTGTGATCTCGAGGTTGC |  |  |  |
| pNK82 | *nifW* | 5′ | F | PbnifN-f2SacI | CATTTAGACGAGCTCTTATGGCGCTG | 1,899 | pNK80, SacI | XhoI |
|  |  |  | R | PbnifW-r4SacI | CAACCTTGAGCTCATAAGGTAGCTG |  |  |  |
| pNK13 | *pflB* | 3′ | F | PbpflB-f7SalI | ACACACCCGATGGTCGACAAAGCAGG | 2,034 | pUCK192^h^, SalI | na |
|  |  |  | R | PbadhE-r2SalI | CATGCCGTTGTCGACGTCTTACTGAG |  |  |  |
|  |  | 5′ | F | Pborf332-f1BglII | ACCGCAGCAGATCTAAGTGAATTCG | 1,902 | BamHI* | KpnI, NdeI |
|  |  |  | R | PbpflB-r5BglII | GAAACGAAGATCTCCGGTGTAAGGTG |  |  |  |
| pNK12 | *nafY* | 3′ | F | PbnifY-f2XbaI | CTGATTCTAGATTGCCATGTCGTG | 1,539 | pUC19Cm2^i^, XbaI, HindIII | na |
|  |  |  | R | PbcyaA-r1HindIII | TCTAAGCTTTGTGCAGCATCCGTGAGC |  |  |  |
|  |  | 5′ | F | PbdppA-f1SacI | TCCGAGCTCTTAAGCAGCTTTCTGATGTG | 1,858 | SacI, BamHI* | SacI, HindIII |
|  |  |  | R | PbnifY-r2BamHI | ATTGGATCCTCGCCTTCTTCTTCACC |  |  |  |

^a^ Mutants were named after the plasmid name (without the first ‘p’). ^†^ intermediate plasmids. ^§^ pNK75 was constructed by connecting the short 5′-part of *nifP* (197 bp) to the first plasmid pNK71 with In-Fusion.

^b^ For homologous recombination with the chromosomal DNA, the plasmids carry 5′- and 3′-parts of the target gene, which were amplified by PCR from the genomic DNA, on both sides of the Km^R^ gene.

^c^ D indicates the direction of the PCR primers; F, forward; and R, reverse.

^d^ Restriction sites are underlined. Additional sequences for In-Fusion are double underlined.

^e^ The bp length of the amplified PCR fragment.

^f^ The plasmid and its restriction sites that were used to insert amplified PCR fragments.

^g^ Restriction enzyme(s) used to linearize the plasmid and introduce them into *L. boryana* cells by electroporation. na, not applicable.

^h^ Tsujimoto et al. (2014)

^i^ A 2.3-kb HincII-EcoRV fragment carrying the *cat* gene (chloramphenicol resistance (Cm^R^) cartridge) of pBR325 was introduced into the EcoRV site of pBluescript II, and then the 1.6-kb BamHI-BclI fragment was cloned into the BamHI site of pUC19 to form pUC19Cm2.

* indicates that the amplified PCR fragment was introduced into the intermediate plasmid shown in the above cell.

**Supplementary Table 2** List of primers used for colony PCR, RT-PCR, and real-time PCR

| Colony PCR/RT-PCR (cycle number) | Mutant | Target gene | Primer | Sequence |
| --- | --- | --- | --- | --- |
| Colony PCR | NK8 | *nifX*/*∆nifX* | PbnifX-f2 | GCTCATTTTGGCTCAGCCAGTAAG |
|  |  |  | Pborf155-r1 | GCTCTATCCTTTCGCTGCATCAGG |
| RT-PCR (24) | – | *nifX* | PbnifX-f2 | GCTCATTTTGGCTCAGCCAGTAAG |
|  |  |  | PbnifX-r2 | GCTGGAGTGCTTTGCGTAACCAC |
| Colony PCR | NK12/NK812 | *nafY*/*∆nafY* | PbnifY-f1 | GTCTTGAATGAAGCAGCACTCAGC |
|  |  |  | PbnifY-r1 | GCTTGCACAACCTTGGCAGCAG |
| RT-PCR (29) | – | *nafY* | PbnifY-f3 | GATGCCAATGCAGGCGACATTGC |
|  |  |  | PbnifY-r3 | CAGTCAGAATCGCTCTTGCTTCACC |
| Colony PCR | NK21 | *nifZ*/*∆nifZ* | PbnifV-f3 | CCTGGAAAGCGATCGTCGGATC |
|  |  |  | Pbhypo1-r3 | GTTACAACAACGCAAAGCCCTATC |
| Colony PCR | NK22 | *nifT*/*∆nifT* | PbnifV-f3 | CCTGGAAAGCGATCGTCGGATC |
|  |  |  | PbnifT-r1Bglll | TCAGGGCGAGATCTGAACGGAGCGAG |
| Colony PCR | NK75 | *nifP*/*∆nifP* | PbnifP-379-f | ACCGTAACCACAGATACAGA |
|  |  |  | Pborf84-r | CTTCGAGTGTGCTGAAACAT |
| Colony PCR | NK91 | *hesA*/*∆hesA* | PbnifW-f2 | TCCCAGAGATTGATGATGCAGAGC |
|  |  |  | PbhesB-r2 | AGGCGAAGTTCTGCTCTTTCCGTC |
| Colony PCR | NK92 | *hesB*/*∆hesB* | PbhesB-f3 | GACGGTTACTTTGACGGAAAGAGC |
|  |  |  | PbfdxB-r1 | TTCAGAGATTCGTGGGTGTAGCAG |
| Colony PCR | NK82 | *nifW*/*∆nifW* | Pborf70-f1 | GATGCCAACGACGACCAGCGTTG |
|  |  |  | PbhesA-r1 | AGTCGTCGCTGTGCATCTTCGTC |
| Colony PCR* | NK13 | *pflB*/*∆pflB* | PbpflB-f5 | AGGCGATCCCACTTGGGTGACTG |
|  |  |  | PbpflA-r1 | TGTCGATCGCGGCTTGAACTTGC |
|  |  |  | KmR-f1 | TGCTGCCTGAGGCTGGACGAC |
| RT-PCR (23) | – | *nifH* | PbnifH-f1 | GCTATCGCGATGTGAAGTGCGTTG |
|  |  |  | PbnifH-r1 | CTTGTGCTTGAGCGCTGTCAGG |
| RT-PCR (24) | – | *nifD* | pPbnifD-f2 | CAAAGACGTTCTAGAGGCATACCCTG |
|  |  |  | pPbnifD-r2 | GTTGTACTCCAACCAGGGAATACC |
| RT-PCR (24) | – | *nifK* | PbnifK-f1 | CGAACTCTTACAGCCTCTACAAGC |
|  |  |  | PbnifK-r1 | GCAGGTTGATCGTTCCCTTGTACC |
| RT-PCR (24) | – | *rpoA* | PbrpoA-f1 | GACCCGACTCAGTACATTGCAACC |
|  |  |  | PbrpoA-r1 | GACTTCCTCGGCAGACTTGGCTC |
| Real-time PCR | *∆nifP*/YFD1 | *nifE* | PbnifE-f4 | CCTCTACAAGATGGGCTTTACCAC |
|  |  |  | PbnifE-r1 | CAGGTGAATTGACAGGCACAACTGG |
| Real-time PCR | *∆nifP*/YFD1 | *nifH* | PbnifH-f1 | GCTATCGCGATGTGAAGTGCGTTG |
|  |  |  | PbnifH-r2 | CTTTACCTTCGCGAATTGGCATTGC |
| Real-time PCR | *∆nifP*/YFD1 | *rnpB* | PbrnpB-f1 | GGACTCCCCAAAACCCAGACTTGC |
|  |  |  | PbrnpB-r1 | CCTTTGTTTCGTCTGACCTTGCTC |

*Since sizes of the PCR products of the wild type and *∆pflB* with two normal primers, PbpflB-f5 and PbpflB-r1, are almost similar, three primers were used in colony PCR. A 2.1 kb PCR fragment is amplified from the wild type copy with PbpflA-r1 and PbpflB-f5, and a 1.7 kb PCR fragment is amplified from the mutant copy with PbpflA-r1 and KmR-f1 (see Supplementary Figure 7).

**References**

Becker, A., Frits-Wolf, K., Kabsch, W., Knappe, J., Schultz, S., and Wagner, A.F.V. (1999) Structure and mechanism of the glycyl radical enzyme pyruvate formate-lyase. *Nature Struct. Mol. Biol.* 6, 969-975.

Pyre, V.E., Tingey, A.P., Robson, R.L., and Moody, P.C.E. (2004) The structure and mechanism of serine acetyltransferase from *Escherichia coli*. *J. Biol. Chem.* 279, 40729-40736.

Rubio, L.M., Singer, S.W., and Ludden, P.W. (2004). Purification and characterization of NafY (Apodinitrogenase γ subunit) from *Azotobacter vinelandii*. *J. Biol. Chem.* 279, 19739-19746.

Tsujimoto, R., Kamiya, N., and Fujita, Y. (2014) Transcriptional regulators ChlR and CnfR are essential for diazotrophic growth in nonheterocystous cyanobacteria. *Proc. Natl. Acad. Sci. U. S. A.* 111, 6762-6767.
